# Editorial: Glial-targeted therapeutics for CNS disease: getting there from here

**DOI:** 10.3389/fncel.2023.1231648

**Published:** 2023-07-03

**Authors:** Malú Gámez Tansey, Ashley S. Harms

**Affiliations:** ^1^Departments of Neuroscience and Neurology, Center for Translational Research in Neurodegenerative Disease and Fixel Institute for Neurological Diseases, University of Florida, Gainesville, FL, United States; ^2^Department of Neurology, University of Alabama at Birmingham, Birmingham, AL, United States

**Keywords:** glia, therapeutic targets, astrocyte, microglia, inflammation, neurodegeneration, neuroinflammation

The original dogma in the neuroscience field was that the central nervous system (CNS) is an immune-privileged site and that glia play a passive role supporting neurons in the CNS; but in the last two decades, glial and brain-resident immune cells have emerged as vital players that function to actively maintain the health of neurons. Therefore, the CNS should be viewed as an immune-specialized compartment which is, in fact, in bidirectional communication with peripheral organs and compartments. Importantly, the same critical functions that glial and immune cells execute in promoting neuronal health can become maladaptive when they are altered in the context of aging and disease. Specifically, a significant body of literature now indicates that glial cells and immune-based mechanisms can actively contribute to disease pathology in a number of different neuroinflammatory and neurodegenerative diseases of the human CNS. The aim of this Research Topic is to discuss several of these emerging pathological mechanisms and consider translational strategies for targeting specific pathways to bring novel therapeutics into clinical development to treat, delay, or prevent these devastating neurodegenerative diseases.

Importantly, the preclinical path to developing such therapeutics and successfully translating them to the clinic remains marked with challenges. In particular, animal models of neuroinflammatory and neurodegenerative CNS disease have been developed, but species differences in immunology (e.g., rodent vs. human) pose significant challenges for understanding disease mechanisms and translating novel therapies. Additionally, challenges remain in identifying cell culture systems predictive of *in vivo* glial cell responses and *in vivo* approaches must further consider the effects that anesthesia can have on glial function in the CNS. Furthermore, many studies of drugs or molecules with therapeutic potential rely solely on acute dosing paradigms and quite often the rescue of pharmacodynamic endpoints is unrelated to disease pathogenesis, rather than also including chronic dosing and endpoints that are more appropriate to disease pathophysiology. Lastly, an additional challenge for immune-based therapeutics is to define whether the CNS and/or peripheral compartments need to be specifically targeted depending on the mechanism being considered as well as the timing of the intervention.

This Research Topic includes studies on preclinical development and clinical translation of novel therapeutics targeting immune and/or glial cell mechanisms to treat CNS disease. Three core themes are covered: (1) Novel therapeutic development for neurodegenerative and neuroinflammatory CNS diseases; (2) Influence of novel glial therapeutics and targets on neuronal function and structure; and (3) Clinical translation strategies for glia-targeted therapeutics. Fu et al., discuss the role of microglia in the blood-retinal barrier (BRB) and retinopathy, raising the possibility that their inflammatory activities could be targeted to treat neovascularization in the BRB (REF). Connor et al., explore the potential for the small-molecule GW5074, a c-Raf inhibitor to increases microglia phagocytic activities around amyloid in the context of Alzheimer's-like pathology (REF). Massenzio et al., describe how inhibition of the Ca^2+^-activated K+ channel (KCa_3_.1) with TRAM-34 in tumor-associated macrophages (TAMs) can promote phenotype switching in the tumor microenvironment in glioma (REF). MacPherson et al., investigate the central and peripheral effects of an obesogenic diet on Alzheimer's-like pathology and the protective effects of targeting soluble tumor necrosis factor (TNF) with XPro1595/pegipanermin, a novel biologic currently in Phase 2 trials in Mild Cognitive Impairment (MCI) and Alzheimer's disease (AD) (REF). Abulseoud et al., review the pre-clinical and clinical evidence supporting the efficacy of Ceftriaxone (CEF) as a novel therapeutic agent for targeting glutamate transporter-1 (GLT-1)-dependent and independent hyper-glutamatergic states such as such as ischemic stroke, amyotrophic lateral sclerosis (ALS), seizure, Huntington's disease (HD), and various aspects of drug use disorders (REF). Pan et al., identify fluoxetine's anti-depressive effects in corticosterone-induced depressive-like disorder are mediated by increasing astrocytic glucose uptake and glycolysis through the glucocorticoid receptor-thioredoxin-interacting protein-glucose transporter-1 (GR-TXNIP-GLUT1) pathway (REF).

## Author contributions

MT and AH drafted and edited the editorial. All authors contributed to the article and approved the submitted version.

